# 2-Cyano-*N*,*N*-dimethyl­acetamide

**DOI:** 10.1107/S1600536812000748

**Published:** 2012-01-18

**Authors:** Shan Liu, Hong-Jun Zhu, Guo-Quan Yu, Gang Du, Liang-Zhong Lv

**Affiliations:** a Nanjing College of Chemical Technology, No. 625, Geguan Road, Luhe, Nanjing 210048, People’s Republic of China; b Department of Applied Chemistry, College of Science, Nanjing University of Technology, Nanjing 210009, People’s Republic of China; c Jiangsu Changqing Agrochemical Co. Ltd, No.1 Jiangling Road, Putou Town, Jiangdu City, Jiangsu 225218, People’s Republic of China

## Abstract

In the crystal structure of the title compound, C_5_H_8_N_2_O, mol­ecules are linked by weak C—H⋯O hydrogen bonds, forming a three-dimensional network.

## Related literature

For uses of 2-cyano-*N*, *N*-dimethyl­acetamide, see: Liu *et al.* (2011 ▶). For the synthesis, see: Liu *et al.* (2011 ▶). For bond-length data, see: Allen *et al.* (1987 ▶).
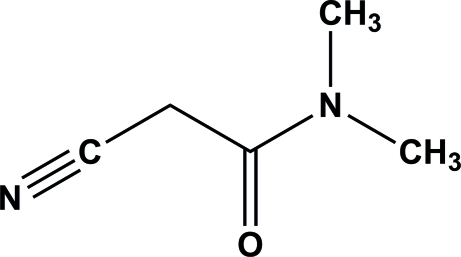


## Experimental

### 

#### Crystal data

C_5_H_8_N_2_O
*M* *_r_* = 112.13
Monoclinic, 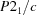

*a* = 4.1690 (8) Å
*b* = 9.3940 (19) Å
*c* = 15.880 (3) Å
β = 92.67 (3)°
*V* = 621.2 (2) Å^3^
*Z* = 4
Mo *K*α radiation
μ = 0.09 mm^−1^
*T* = 298 K
0.30 × 0.20 × 0.10 mm

#### Data collection

Enraf–Nonius CAD-4 diffractometer
Absorption correction: ψ scan (North et al., 1968) ▶ *T*_min_ = 0.975, *T*_max_ = 0.991
1294 measured reflections
1129 independent reflections
666 reflections with *I* > 2σ(*I*)
*R*_int_ = 0.051
3 standard reflections every 200 reflections intensity decay: 1%

#### Refinement

*R*[*F* ^2^ > 2σ(*F* ^2^)] = 0.068
*wR*(*F* ^2^) = 0.167
*S* = 1.01
1129 reflections
73 parameters
H-atom parameters constrained
Δρ_max_ = 0.22 e Å^−3^
Δρ_min_ = −0.18 e Å^−3^

### 

Data collection: *CAD-4 Software* (Enraf–Nonius, 1985) ▶; cell refinement: *CAD-4 Software* ▶; data reduction: *XCAD4* (Harms & Wocadlo, 1995 ▶); program(s) used to solve structure: *SHELXS97* (Sheldrick, 2008 ▶); program(s) used to refine structure: *SHELXL97* (Sheldrick, 2008 ▶); molecular graphics: *SHELXTL* (Sheldrick, 2008 ▶); software used to prepare material for publication: *SHELXTL*.

## Supplementary Material

Crystal structure: contains datablock(s) I, global, n1. DOI: 10.1107/S1600536812000748/lx2222sup1.cif

Structure factors: contains datablock(s) I. DOI: 10.1107/S1600536812000748/lx2222Isup2.hkl

Supplementary material file. DOI: 10.1107/S1600536812000748/lx2222Isup3.cml

Additional supplementary materials:  crystallographic information; 3D view; checkCIF report

## Figures and Tables

**Table 1:** Hydrogen-bond geometry (Å, °)

*D*—H⋯*A*	*D*—H	H⋯*A*	*D*⋯*A*	*D*—H⋯*A*
C4—H4*A*⋯O^i^	0.97	2.38	3.300 (3)	159
C4—H4*B*⋯O^ii^	0.97	2.41	3.141 (3)	132

Symmetry codes: (i) 
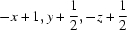
; (ii) 
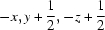
.

**Fig. 1.**
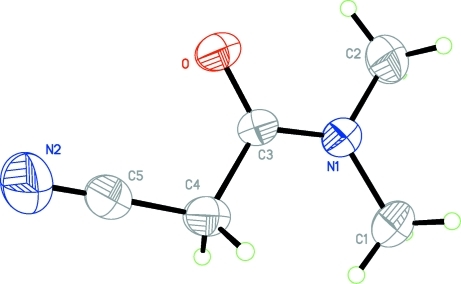
The molecular structure of the title compound with the atom numbering scheme. Displacement ellipsoids are drawn at the 40% probability level. H atoms are presented as small spheres of arbitrary radius.

**Fig. 2.**
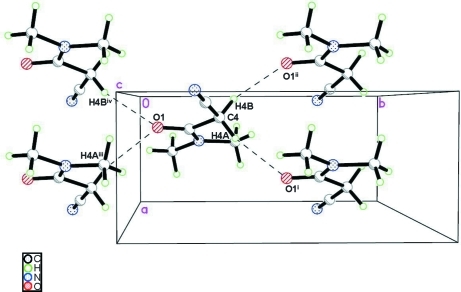
A view of the C–H···O hydrogen bonds (dotted lines) in the crystal structure of the title compound. [Symmter codes: (i) - x + 1, y + 1/2 , - z + 1/2; (ii) - x, y + 1/2, - z + 1/2; (iii) - x + 1, y - 1/2, - z + 1/2; (iv) - x , y -1/2, - z -1/2.]

## supplementary crystallographic information

### Comment

2-Cyano-*N*, *N*-dimethylacetamide is an important intermediate used to synthesize the herbicide of nicosulfuron (Liu *et al.*, 2011). We report here the crystal structure of the title compound (Fig. 1).

In the title molecule, bond lengths (Allen *et al.* , 1987) and angles are within normal ranges. In the crystal packing (Fig. 2), molecules are linked by weak intermolecular C–H···O hydrogen bonds (see, Table 1).

### Experimental

2-Cyano-*N*,*N*-dimethylacetamide was prepared by the method reported in literature (Liu *et al.*, 2011). Single crystals were obtained by dissolving 2-Cyano-*N*, *N*-dimethylacetamide (0.50 g, 4.46 mmol) in ethyl acetate (30 ml) and evaporating the solvent slowly at room temperature for about 7 d.

### Refinement

H atoms were positioned geometrically, with O–H = 0.82 and C–H = 0.93Å for aromatic H, and constrained to ride on their parent atoms, with *U*_iso_(H) = *xU*_eq_(C/O), where *x* = 1.2 for aromatic H and *x* = 1.5 for other H.

### Crystal data

**Table:**

C_5_H_8_N_2_O	*F*(000) = 240
*M**_r_* = 112.13	*D*_x_ = 1.199 Mg m^−^^3^
Monoclinic, *P*2_1_/*c*	Mo *K*α radiation, λ = 0.71073 Å
Hall symbol: -P 2ybc	Cell parameters from 25 reflections
*a* = 4.1690 (8) Å	θ = 9–13°
*b* = 9.3940 (19) Å	µ = 0.09 mm^−^^1^
*c* = 15.880 (3) Å	*T* = 298 K
β = 92.67 (3)°	Block, brown
*V* = 621.2 (2) Å^3^	0.30 × 0.20 × 0.10 mm
*Z* = 4	

### Data collection

**Table:**

Enraf–Nonius CAD-4 diffractometer	666 reflections with *I* > 2σ(*I*)
Radiation source: fine-focus sealed tube	*R*_int_ = 0.051
graphite	θ_max_ = 25.4°, θ_min_ = 2.5°
ω/2θ scans	*h* = 0→5
Absorption correction: ψ scan (North et al., 1968)	*k* = 0→11
*T*_min_ = 0.975, *T*_max_ = 0.991	*l* = −19→19
1294 measured reflections	3 standard reflections every 200 reflections
1129 independent reflections	intensity decay: 1%

### Refinement

**Table:**

Refinement on *F*^2^	Primary atom site location: structure-invariant direct methods
Least-squares matrix: full	Secondary atom site location: difference Fourier map
*R*[*F*^2^ > 2σ(*F*^2^)] = 0.068	Hydrogen site location: difference Fourier map
*wR*(*F*^2^) = 0.167	H-atom parameters constrained
*S* = 1.01	*w* = 1/[σ^2^(*F*_o_^2^) + (0.09*P*)^2^ + 0.*P*] where *P* = (*F*_o_^2^ + 2*F*_c_^2^)/3
1129 reflections	(Δ/σ)_max_ < 0.001
73 parameters	Δρ_max_ = 0.22 e Å^−^^3^
0 restraints	Δρ_min_ = −0.18 e Å^−^^3^

### Special details

**Table:**

Geometry. All e.s.d.'s (except the e.s.d. in the dihedral angle between two l.s. planes) are estimated using the full covariance matrix. The cell e.s.d.'s are taken into account individually in the estimation of e.s.d.'s in distances, angles and torsion angles; correlations between e.s.d.'s in cell parameters are only used when they are defined by crystal symmetry. An approximate (isotropic) treatment of cell e.s.d.'s is used for estimating e.s.d.'s involving l.s. planes.
Refinement. Refinement of *F*^2^ against ALL reflections. The weighted *R*-factor *wR* and goodness of fit *S* are based on *F*^2^, conventional *R*-factors *R* are based on *F*, with *F* set to zero for negative *F*^2^. The threshold expression of *F*^2^ > σ(*F*^2^) is used only for calculating *R*-factors(gt) *etc*. and is not relevant to the choice of reflections for refinement. *R*-factors based on *F*^2^ are statistically about twice as large as those based on *F*, and *R*- factors based on ALL data will be even larger.

### Fractional atomic coordinates and isotropic or equivalent isotropic displacement parameters (Å^2^)

**Table:**

	*x*	*y*	*z*	*U*_iso_*/*U*_eq_	
O	0.2785 (4)	0.08459 (15)	0.22552 (10)	0.0789 (6)	
C4	0.1898 (6)	0.3244 (2)	0.26152 (15)	0.0703 (7)	
H4A	0.3718	0.3851	0.2766	0.084*	
H4B	0.0286	0.3824	0.2319	0.084*	
C3	0.2962 (6)	0.2083 (2)	0.20353 (15)	0.0617 (6)	
N1	0.4071 (5)	0.2461 (2)	0.13046 (13)	0.0747 (7)	
C5	0.0598 (7)	0.2697 (3)	0.33702 (18)	0.0758 (8)	
C2	0.5089 (8)	0.1366 (3)	0.07292 (18)	0.0956 (9)	
H2A	0.4964	0.0451	0.0995	0.143*	
H2B	0.7263	0.1545	0.0585	0.143*	
H2C	0.3715	0.1378	0.0227	0.143*	
N2	−0.0412 (8)	0.2295 (3)	0.39639 (19)	0.1157 (10)	
C1	0.4201 (9)	0.3936 (3)	0.09947 (19)	0.1010 (11)	
H1A	0.3514	0.4574	0.1423	0.151*	
H1B	0.2812	0.4033	0.0499	0.151*	
H1C	0.6363	0.4163	0.0860	0.151*	

### Atomic displacement parameters (Å^2^)

**Table:**

	*U*^11^	*U*^22^	*U*^33^	*U*^12^	*U*^13^	*U*^23^
O	0.1110 (14)	0.0480 (10)	0.0791 (12)	−0.0014 (10)	0.0192 (10)	0.0048 (8)
C4	0.0821 (16)	0.0537 (14)	0.0757 (17)	0.0009 (13)	0.0097 (13)	−0.0052 (11)
C3	0.0797 (16)	0.0448 (12)	0.0604 (14)	−0.0005 (12)	0.0003 (11)	0.0023 (10)
N1	0.1086 (18)	0.0552 (11)	0.0609 (12)	−0.0038 (12)	0.0102 (11)	0.0037 (9)
C5	0.0924 (18)	0.0651 (15)	0.0708 (18)	−0.0047 (15)	0.0125 (14)	−0.0105 (13)
C2	0.128 (2)	0.093 (2)	0.0672 (17)	0.0092 (19)	0.0192 (16)	−0.0067 (15)
N2	0.141 (2)	0.116 (2)	0.094 (2)	−0.0157 (19)	0.0406 (18)	−0.0064 (17)
C1	0.157 (3)	0.0709 (17)	0.0753 (19)	−0.015 (2)	0.0080 (18)	0.0182 (14)

### Geometric parameters (Å, °)

**Table:**

O—C3	1.217 (2)	C5—N2	1.116 (3)
C4—C5	1.434 (4)	C2—H2A	0.9600
C4—C3	1.507 (3)	C2—H2B	0.9600
C4—H4A	0.9700	C2—H2C	0.9600
C4—H4B	0.9700	C1—H1A	0.9600
C3—N1	1.318 (3)	C1—H1B	0.9600
N1—C2	1.453 (3)	C1—H1C	0.9600
N1—C1	1.472 (3)		
			
C5—C4—C3	112.6 (2)	N1—C2—H2A	109.5
C5—C4—H4A	109.1	N1—C2—H2B	109.5
C3—C4—H4A	109.1	H2A—C2—H2B	109.5
C5—C4—H4B	109.1	N1—C2—H2C	109.5
C3—C4—H4B	109.1	H2A—C2—H2C	109.5
H4A—C4—H4B	107.8	H2B—C2—H2C	109.5
O—C3—N1	122.6 (2)	N1—C1—H1A	109.5
O—C3—C4	119.5 (2)	N1—C1—H1B	109.5
N1—C3—C4	117.94 (19)	H1A—C1—H1B	109.5
C3—N1—C2	119.2 (2)	N1—C1—H1C	109.5
C3—N1—C1	124.6 (2)	H1A—C1—H1C	109.5
C2—N1—C1	116.1 (2)	H1B—C1—H1C	109.5
N2—C5—C4	178.7 (3)		
			
C5—C4—C3—O	−3.2 (3)	O—C3—N1—C1	177.2 (3)
C5—C4—C3—N1	177.1 (2)	C4—C3—N1—C1	−3.1 (4)
O—C3—N1—C2	0.8 (4)	C3—C4—C5—N2	167 (15)
C4—C3—N1—C2	−179.5 (2)		

### Hydrogen-bond geometry (Å, °)

**Table:**

*D*—H···*A*	*D*—H	H···*A*	*D*···*A*	*D*—H···*A*
C4—H4A···O^i^	0.97	2.38	3.300 (3)	159.
C4—H4B···O^ii^	0.97	2.41	3.141 (3)	132.

Symmetry codes: (i) −*x*+1, *y*+1/2, −*z*+1/2; (ii) −*x*, *y*+1/2, −*z*+1/2.
